# Cold Water Swimming—Benefits and Risks: A Narrative Review

**DOI:** 10.3390/ijerph17238984

**Published:** 2020-12-02

**Authors:** Beat Knechtle, Zbigniew Waśkiewicz, Caio Victor Sousa, Lee Hill, Pantelis T. Nikolaidis

**Affiliations:** 1 Medbase St. Gallen Am Vadianplatz, 9000 St. Gallen, Switzerland; beat.knechtle@hispeed.ch; 2 Institute of Sport Science, Jerzy Kukuczka Academy of Physical Education, 40-065 Katowice, Poland; z.waskiewicz@awf.katowice.pl; 3 Department of Sports Medicine and Medical Rehabilitation Moscow, Sechenov First Moscow State Medical University, 19c1 Moscow, Russia; 4 Bouve College of Health Sciences, Northeastern University, Boston, MA 02115, USA; cvsousa89@gmail.com; 5 Division of Gastroenterology & Nutrition, Department of Pediatrics, McMaster University, Hamilton, ON L8N 3Z5, Canada; hilll14@mcmaster.ca; 6 School of Health and Caring Sciences, University of West Attica, 12243 Athens, Greece

**Keywords:** hypothermia, endurance sports, adaptation, heart, immune system

## Abstract

Cold water swimming (winter or ice swimming) has a long tradition in northern countries. Until a few years ago, ice swimming was practiced by very few extreme athletes. For some years now, ice swimming has been held as competitions in ice-cold water (colder than 5 °C). The aim of this overview is to present the current status of benefits and risks for swimming in cold water. When cold water swimming is practiced by experienced people with good health in a regular, graded and adjusted mode, it appears to bring health benefits. However, there is a risk of death in unfamiliar people, either due to the initial neurogenic cold shock response or due to a progressive decrease in swimming efficiency or hypothermia.

## 1. Introduction

**Figure 1:**
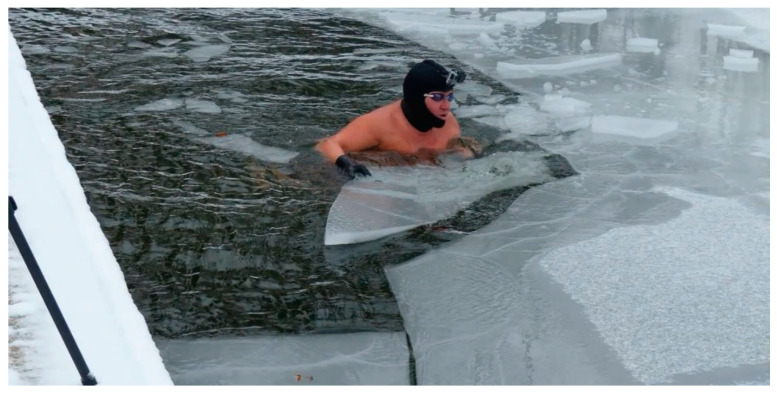
Ice swimmer in the preparation of his pool for training (Picture with permission of the athlete).

Cold water swimming—also known as winter swimming or ice swimming—describes swimming outdoors (lake, river, sea, swimming pool, etc.) mainly during the winter or in the colder and polar regions [1]. This special form of endurance sport is becoming increasingly popular. Cold water swimming can be used as a general umbrella term for swimming in cold to ice-cold water. Winter swimming specifically implies that it must be winter. In colder countries, it can be synonymous with ice swimming when the water is frozen over because ice swimming explicitly requires the ice to break (Figure 1). In recent years, ice swimming (in water below 5 °C) has evolved into an all year-round sport [2], with many swimmers participating and competing regularly in both local and international events. 

Several studies have suggested that cold water swimming has a wide variety of health benefits [3], including changes in hematological [4] and endocrine function [5,6], fewer upper respiratory tract infections [7], amelioration of mood disorders [8] and general well-being [9]. Although chronic exposure to colder water temperatures has been shown to be beneficial to one’s health, several studies have outlined the potential risks [10,11,12,13]. Therefore, the primary purpose of this review is to outline the potential benefits and risks of cold water swimming. 

## 2. Methods

A narrative review was performed as previously described [14] between 3 and 14 August 2020 using the databases PUBMED and SCOPUS with the terms ‘cold water swim (ming)’ or ‘ice water swim (ming)’ or ‘winter swim (ming)’. The term ‘cold water swim’ was leading to 822 publications, the term ‘cold water swimming’ to 742 publications. When looking for ‘ice water swim’, we obtained 144 publications, and 131 publications with the term ‘ice water swimming’. For ‘winter swim’, we obtained 340 publications and for ‘winter swimming’ a total of 314 publications. Case reports and field studies were examined for their clinical and practical relevance; animal and laboratory studies were not taken into account. Only studies with practical and clinical relevance were considered. 

We organize the review, starting with the historical aspect of winter swimming and then turning to current times, where ice swimming has become a new sports discipline. Since this sport discipline is potentially fatal, we looked for benefits and risks to prepare and compete in these races.

## 3. Historical Aspects and Definitions

Since antiquity, cold water immersion has been regarded with both reverence and fear. As far back as 450 BC, the ancient Greek historian Herodotus described the unfortunate expedition of the Persian General Mardonius, noting that ‘…those who could not swim perished from that cause, others from the cold’ [15]. Later, in December 1790, Dr. James Currie became interested in the physiological effects of hypothermia after helplessly observing three crew members of a stranded American sailing ship who fell and drowned in the 5 °C cold sea [16]. This experience prompted Dr. Currie to carry out the first recorded experiments on the effects of cold-water immersion and hypothermia on humans [17], ultimately leading to the discovery of afterdrop. The modern age of open water swimming, as opposed to swimming, probably began on 3 May 1810, when Lord Byron swam several miles across the Dardanelles (Hellespont) from Europe to Asia [18]. 

There is no strict definition of ‘cold water’. However, given that the majority of observable dangerous reactions to cold water appears to peak when immersed between 15 and 10 °C, it is reasonable to say that cold water is defined as water with a temperature of <15 °C [19]. However, the thermoneutral water temperature for a resting undressed person is ~35 °C, so that people may experience a decrease in the core body temperature over time when immersed in water below this temperature. The corresponding temperature for long-distance swimmers’ training is ~25 °C [20].

## 4. Winter Swimming

In certain northern countries, such as Finland, Poland, Russia, Norway, Sweden, Denmark, Estonia, Lithuania, the Czech Republic and Latvia, cold water swimming is practiced regularly in the sense of winter swimming. In Eastern Europe and Russia, winter swimming is part of the celebration of Epiphany [21]. Naturally, many field studies investigating the influence of cold water swimming on the body come from these northern countries on various topics such as adaptation to the cold [22], changes in lipid metabolism [23,24], adjustments to hematological values [25,26], effects on the immune system [27,28,29,30] and the hormones [5,31] or aspects of thermoregulation [32,33,34,35]. Events in which large numbers of people swim over a relatively short distance in cold water in winter can also be called classic winter swimming.

## 5. Beginnings of the Sport of Ice Swimming

Ice swimming is a unique form of cold-water or winter swimming. As a rule, ice swimming is carried out in an environment where freezing temperatures prevail regardless of the season, such as at the north or south pole. Ice swimming is specifically practiced by extreme athletes, where the American Lynne Cox [36] and the Briton Lewis Gordon Pugh [37] are considered among the best known and most extreme ice swimmers in the world. 

In her early days, Lynne Cox successfully completed many official crossings. These include the Santa Catalina Canal in California, the English Channel from England to France, the Cook Strait between the north and south islands of New Zealand, the Magellan Strait on the southern tip of Chile, the Øresund between Denmark and Sweden, the Skagerrak between Sweden and Norway, Lake Baikal and Lake Titicaca. Later, she started swimming in ice-cold water. In 1987, she crossed the Bering Strait from the Island of Little Diomede in Alaska to the Island of Big Diomede (then the Soviet Union, now Russia) at a water temperature of ~4 °C. In 2002, she swam in the ice-cold waters of the Antarctic for around 25 min, reaching around 1.7 km [36].

Lewis Gordon Pugh made the first attempt in July 2007 to swim a long distance in ice-cold water as close as possible to the geographical North Pole [38]. He managed to swim a distance of 1 km for 18:50 min:s in −1.7 °C cold water at an open point in the ice at the North Pole. On 22 May 2010, Pugh became the first person to swim through Lake Pumori, the lake of the Khumbu Glacier at the foot of Mount Everest at 5300 meters above sea level. After a two-week climb to the Mount Everest base camp, he managed to swim the distance of 1 km in 2 °C cold water in 22:51 min:s [38]. In 2005, Pugh set the record for the most northern swim course ever swum when he circled Verenkenhuken, the most northern cape of the Island of Spitsbergen [38]. Five months later, he broke Lynne Cox’s record for the southernmost swim course ever swum when he circled Petermann Island (65° south latitude) in Antarctica [38].

**Figure 2:**
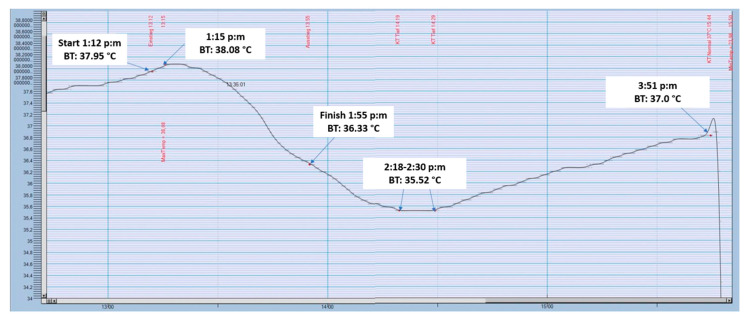
Body core temperature before, during and after an ‘Ice Mile’ (data provided by the athlete with permission). BT: Body temperature.

**Figure 3:**
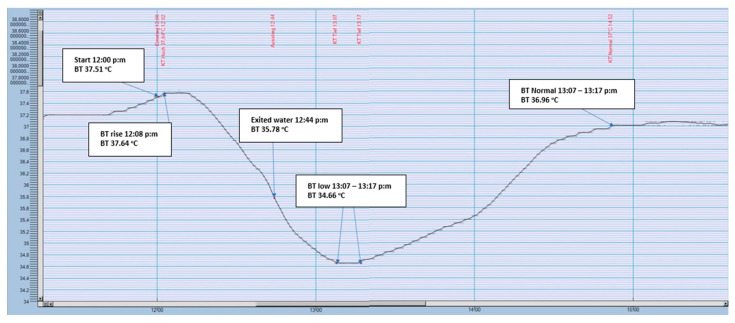
Body core temperature before, during and after an ‘Ice Mile’ (data provided by the athlete with his permission).

South African sports scientist Timothy David Noakes from the University of Cape Town was present in both world record attempts. His research has shown that Pugh’s ability to raise his body temperature by 2 °C enabled him to survive the cold water. His specialist term for this, the ‘anticipatory thermo-genesis’, is a process that has never been registered with any other person [39]. Recently, this increase in core body temperature when immersed in ice-cold water was confirmed several times by German ice swimmer Bruno ‘Orca’ Dobelmann. Observing Dobelmann’s many documented training sessions [40] and under competition conditions, [41] we are able to observe increases in core body temperature immediately after starting swimming in ice-cold water. As Figure 2 and Figure 3 show in the context of repeated so-called ‘ice miles’, this increase was in the range of fractions of a degree and not exactly 2 °C as reported by Noakes and Pugh. However, the type of thermal probe (i.e., sensitivity) and the location of the probe (i.e., stomach, rectum) may have influenced the measurements. In both swimmers, however, body core temperature was measured in the rectum. The so-called ‘anticipatory thermo-genesis’ should, therefore, be a normal physiological reaction in a trained ice swimmer when immersed in ice-cold water [40,41].

## 6. Ice Swimming as a Competition

Since 2009, there have been official ice swimming competitions [42]. The International Ice Swimming Association (IISA) [2] was founded on 1 July 2009 by South African ice swimmer Ram Barkai in Cape Town, South Africa. Barkai previously swam 1.43 miles (2.3 km) in 43:00 min: s at 4 °C water temperature in Zurich accompanied by a boat on 31 January 2009 [43]. This event is considered as the beginning of the so-called ice miles movement.

The officially recognized ‘Ice Mile’ (1608 m) is held in water with a maximum of 5 °C whereby a swimmer is only permitted the use of swimming goggles, swimming cap, and swimwear [41]. In 2014, the 1 km distance was introduced as an official distance in addition to the traditional ‘Ice Mile’ [42].

Ice swimmers are required to swim the official ‘Ice Miles’ on their own [2], whereby temperatures of water and air must be officially measured and recorded by an accompanying person [41]. There are now official competitions for ice or winter swimming across various shorter distances and disciplines. Championships at the national level [44] and continental and world championships are also held [1]. Official winter swimmers do not swim with wetsuits or other thermal protection, but only with standard swimwear as mentioned above. International ice and winter swimming competitions occur around the world, with two of the larger organizing bodies being the IISA [2] and the IWSA [1]. Both organizations have similar competition policies, including water temperatures below 5 °C, a 25-meter pool that is often cut out of frozen water, and swimmers, whose equipment is limited to swimming goggles, a regular swimsuit, and a latex or silicone cap. Wearing a wetsuit is not permitted. Surprisingly, with this type of competitive swimming, the water temperature does not correlate with the competition time for either men or women [42]. In addition to the official ice and winter swimming, there are so-called polar bear plunge events in many places in North America and Western Europe to celebrate New Year, although the participants are not expected to swim, and usually, most do not swim [45]. 

## 7. Effects of Cold Water Swimming

Since ice swimming is of increasing popularity as sports discipline, we need to consider both the risks and benefits of cold water swimming. We need, however, to consider that swimming in cold water as an athlete is different to immersion in cold water for non-athletes. Athletes compete at rather high intensity for several minutes while non-athletes remain for a few minutes without physical activity.

Cold water swimming is a very stressful physiological condition where the entire body is exposed to cold water. Chilled water swimmers, however, through chronic exposure to the cold water environment, are able to reach different degrees of adaptation to the cold. The question arises whether this type of sport has health benefits or may have more harmful effects. As a form of endurance exercise, cold water swimming, even if swimming in cold water is more strenuous, can increase tolerance to stress factors and cause hardening [46]. Siems et al. [46] showed in winter swimmers that intensive short-term whole-body cold exposure induced an oxidative stress. Compared to controls, the baseline concentration of important components of the antioxidative defense system (e.g., superoxide dismutase and catalase) were higher in the winter swimmers. When cold water swimming is practiced by people with good general health in a regular, graded (go with the season) and adjusted mode, it appears to bring some health benefits [10,47]. On the other hand, there is a risk of death if unfamiliar or inadequately adapted, either due to the initial neurogenic cold shock response or due to a progressive decrease in swimming efficiency or progressive hypothermia [10].

In addition, people with obvious or as yet unrecognized cardiovascular pathologies can be more prone to adverse effects because they cause arrhythmias and acute cardiovascular events that can pose a significant health risk. Therefore, a step-by-step strategy is recommended both to start and to build and expand this activity, on the one hand, to promote and maintain acclimatization, to protect against possible risks of cold-water exposure, and possibly to take advantage of the promising health benefits [10,47].

The first claims for the health benefits of cold water swimming date back to 400 BC. According to Hippocrates, water therapy relieved fatigue, and later, Thomas Jefferson reportedly used a cold foot bath every morning for six decades to keep him healthy [15]. It is believed that these health benefits are a result of the physiological reactions and biochemical milieu caused by exposure to cold water [9,48]. Physiological changes occur acutely during cold water swimming, with repeated cold-water swimming developing adaptations that can also affect health.

In the Middle Ages, swimming was not a skill people possessed as it was believed that if they were doomed to hell, they would not be able to cross the Styx River [49]. However, in 1538, the first book introducing swimming and “the human stroke” was written by Wynmann in an attempt to reduce the number of drowning people [15]. Later in 1750, published work began to emerge that recommended swimming and drinking sea water to treat a range of diseases [50,51], with winter being the best time to do this activity. Bathing by the sea peaked in popularity in by the late 18th century when the bathing suit and the purported ‘bathing machine’ were developed [15]. This led to the explosive growth of whole communities and seaside resorts who touted the assumed health benefits of swimming in the sea [15]. Ostensibly, the increased popularity of swimming led to the introduction of lifeguards on the beach [52] as more people sought to the associated health benefits of swimming. Although there are certain risks associated with ice swimming, scientific studies also provide information on the health benefits of cold-water swimming. Various aspects were described such as influence on the cardiovascular system, psychological and immunological aspects (Table 1).

## 8. Cardiovascular and Endocrine System

Several studies have described a positive effect on the cardiovascular system and cardiovascular risk factors. Cold water swimming appears to have a positive impact on cardiovascular risk factors such as lipid profile [23,24,56] or blood pressure [53].

Various hormones such as catecholamines, insulin, Thyroid-stimulating hormone (TSH), Adrenocorticotropic hormone (ACTH), and cortisol also react to the cold stress [11,55,63]. As a form of endurance training, winter swimming—even if it is more strenuous to swim in cold water—can improve adaption to stress. In a field study with 34 middle-aged cold-water swimmers (48–68 years old), different values of lipid metabolism were determined at the beginning (October), in the middle (January) and after the season (April) of winter swimming [24]. There was a decrease in triglycerides between January and April, a lower concentration of homocysteine (high levels are linked to the early development of heart disease) between October and January and between October and April. The decrease in homocysteine was more pronounced in women than in men [24]. These changes were most probably also due to the fact that these swimmers were active, not sedentary. Unfortunately, no control group was investigated.

Cold water swimming seems to have a positive effect on insulin metabolism, although here too, the effect appears to be sex-specific [3,56]. In a field study, 30 cold water swimmers were examined for six months with regard to body composition and insulin sensitivity [3]. The chilled water swimmers were overweight compared to a control group and had a higher percentage of body fat with differences between the sexes. For female and swimmers with lower body fat percentage, there was an increased insulin sensitivity as well as a reduction in insulin secretion and resistance [3].

Swimming in cold water also affects other hormones, such as ACTH and catecholamines [5,58]. As such, it was found that if swimmers participated in winter swimming three times a week at water temperatures of 0–3 °C for 12 weeks, there was an increase in ACTH and cortisol as well as norepinephrine [58]. Water immersions were 20 s per week for 3 winter months in water of a temperature of 0–2 °C.

It is believed that the increase in norepinephrine may lead to reduced pain perception, such as with whole-body cold therapy or with ice swimming [58]. In contrast, regular three-month winter swimming resulted in a decrease in the concentration of catecholamines when measured immediately after immersion. It was concluded that adaptation through habitual exposure to the cold of winter swimming weakened the physiological response and inhibited the rise of the catecholamines [5].

## 9. Influence on the Psyche

Swimming in ice-cold water has also been shown to have a positive effect on the mental side of humans [9,12,64] and can even be anti-depressive [8]. Regular winter swimming led to an improvement in general well-being in swimmers who suffered from rheumatism, fibromyalgia, or asthma [9]. A case report described a 24-year-old woman with symptoms of severe depression and anxiety [8]. The patient had been treated since the age of 17, and the symptoms did not respond to conventional therapies, including Fluoxetine or Citalopram. After the birth of her daughter, she wanted to be free of medication and symptoms. For this purpose, a novel intervention comprising of a weekly program involving cold water swimming was developed. This resulted in an immediate improvement in mood after each swim and a sustained and gradual reduction in the symptoms of depression. The intervention ultimately led to a reduction in medication use and then finally to a discontinuation of the medication. After one year of therapy with cold water swimming, she was medication-free [8]. Due to the increase in catecholamines, cold water swimming could be a treatment for depression as it activates the sympathetic nervous system and increases the concentration of norepinephrine and β-endorphin [59]. Standard exercise in a thermoneutral environment would lead most probably to the same effect and would be much easier to perform.

## 10. Immunological Aspects

There is rising evidence that winter swimmers are more resistant to certain illnesses and infections, experiencing them less frequently and more mildly [65]. The incidence of infectious diseases of the upper respiratory tract is 40% lower in winter swimmers compared to a control group [66]. Furthermore, it has been shown that swimming in cold water has an impact on immune-specific hematology [29,67]. Anecdotally, cold water swimmers state that they suffer fewer and milder infections from regular swimming in cold water [65]. Improved immune response and function is biologically plausible primarily through the release of stress hormones [31,68] in response to cold exposure. Dhabhar [60] argued that short-term physiological stress such as cold exposure prepares the immune system to fight infections. The study of the effects of cold water swimming on the function of the immune system (especially leukocytes and immunoglobulins) has led to contrasting results. This is possibly due to the majority of studies examining individuals and study protocols of unfamiliar people who take a short bath in ice-cold water [69] longer static cold water swimming (stay in the cold water without moving) [27] and experienced long-distance swimmers who trained for 8 h (dynamic cold water swimming) [62] were very different.

If cold water swimming has a positive effect on the immune function, then there should be observable changes in the immune system markers and actual health should improve over the course of an acclimatization program. Ideally, studies should focus on chilled water swimmers who participate in regular cold water training and thus would yield the most robust values. However, there may be differences in response to static cold-water swimming since exercise and cold can both induce increased physiological stress, and their combined effects can exceed the individual effect of each state [61]. In a study by Jansky et al. [11], the immune system’s reactions to static cold-water swimming was investigated through study participants being initially immersed in cold water and then, followed by repeating cold water swimming three times a week over six weeks. The subjects underwent regular winter swimming at least once a week, for 2 to 10 min, at the natural water temperature (6.8 °C (October 1992) to 2.0 °C (January 1993)) in the southern Baltic Sea. It was seen that the adjustment changes both the number of leukocytes at rest and their response to static cold-water swimming. However, these changes were minor and of uncertain importance, and repeated cold-water swimming did not change the response of the immunoglobulins [27]. Furthermore, Brazaitis et al. [28] investigated the response to being immersed intermittently in cold water. Cold stress was induced using intermittent immersion in bath water at 14 °C. It was observed that participants demonstrated different rates of core temperature cooling. Specifically, those who cooled slower showed signs of leukocytosis. However, responses to static cold-water swimming seem to be heavily influenced by the study protocol and participants. The difference in leukocytosis between those who cooled down quickly or slowly could potentially be attributed to the fact that people who cool down more slowly were immersed for a total of 120 min, whereas people who cooled down faster were immersed for an average of 96 min. The use of alternating cold-water swimming and reheating could also have complicated the physiological response as well. It also seems that the extent of leukocytosis could correspond to the strength and duration of the stress. Jansky et al. [27] found no increase in neutrophils after 60 min in water at 14 °C, while Brazaitis et al. [28] demonstrated an increase of 55% after a total of 120 min in 14 °C cold water with periodic reheating. Within ~1 min of leaving the bath, the volunteer was towel dried and temperatures were measured.

It is interesting to note that the ice swimmer only spends a few minutes immersed in cold water, however, the short exposure duration is still sufficient to illicit a measurable physiological response. For example, blood tests performed immediately before and after a 150 m winter swim at 6 °C showed that the leukocytes (neutrophil granulocytes, lymphocytes and monocytes) increased significantly in the blood due to the cold, so that protection against inflammation and respiratory infections can occur [67]. Another study also showed an increase in leukocytes and monocytes, which was seen as a sign of an improvement in the body’s response to stress [29]. However, the clinical significance of these findings is still uncertain. Short-term leukocytosis is caused by leukocytes that leave organs such as the spleen in response to the increase in catecholamines and cortisol in order to be prepared for defense [60]. The most important part of this short-term reaction is a subsequent decrease in the number of leukocytes in the blood when they reach tissues such as the skin [60]. This has not been explicitly investigated in the context of cold-water swimming, but Yeager et al. [70] found that monocytes and neutrophils migrated in response to a concentration of cortisol that is equivalent to that released during acute stress.

However, it is difficult to measure in vivo immune function adequately, therefore upper respiratory tract infection is often a useful measure as it is a very common infection that affects both congenital and acquired elements [71]. Dugué and Leppänen [69] found that trained cold water swimmers had a higher concentration of certain leukocytes than those who were not cold acclimatized. The authors also examined the reactions of both groups to a brief immersion in ice-cold water. However, since this happened after a sauna use, it is impossible to separate the effects of the two temperature ranges. Furthermore, this was the only study that specifically looked at the cold water immersion effects in men and women, separately. However, the small number of participants complicates the meaningfulness of these results. Interestingly, Kormanovski et al. [62] is the only study to have documented the incidence of actual illness. The authors examined 15 experienced long-distance swimmers over a period of six months. Seven swimmers in the group completed three long-distance swims, once at 6 h (in month 1) and twice at 8 h (in months 3 and 6), while the other swimmers rested and served as a control group. Differences between the group of long-distance swimmers and the control group were found with regard to the reaction of the leukocytes and immunoglobulins, both over the entire investigation period and over the period of the long-distance swimming. The heavy training load may have led to a slight reduction in leukocytes in the long-distance swimmer group in the stress-free phase, but the load caused noticeable increases. The number of granulocytes increased almost fourfold during the 8 h swim. The group of long-distance swimmers showed a significant decrease in the concentration of serum immunoglobulins and IgA (immunoglobulin A) in saliva (sIgA, secretory immunoglobulin A) during the training period, whereas this was not the case for control swimmers. SIgA decreased markedly during all three periods of long-distance swimming, but remained unchanged in the control swimmers, whereas the concentration of the serum immunoglobulins showed no clear pattern in any group. This suggests that there is a connection between the extent of stress and the concentration of leukocytes [60,67]. Long-distance swimmers in the study by Kormanovski et al. [62] showed no significant change in neutrophils after 1 h, but after 2 h the numbers had increased by ~50%, with a quadrupling after 8 h. The non-acclimatized swimmers in the study by Lombardi et al. [67] showed the fastest response with a 38% increase in the number of neutrophils after a race over 150 m. However, this was compared to the previous day, so part of the increase could be due to the psychological stress on race day. All of the studies mentioned report higher leukocyte counts in cold water swimmers, but it is important to emphasize that it is not known whether these higher numbers reflect in the body or a redistribution between different tissues. Finally, it is crucial that the level of cold water acclimatization should be clearly defined as habitual exposure, as discussed previously, heavily influences the magnitude of physiological reaction. The swimmers in the study of Kormanovski et al. [62] were very well trained, whereas those of Lombardi et al. [67] were not acclimatized.

## 11. Upper Respiratory Tract Infections

As mentioned previously, upper respiratory tract infections are a useful proxy measure of the in vivo immune function and several studies have also looked at the prevalence of upper respiratory tract infections in cold water swimmers. In the first of these studies, Brenke [65] surveyed 85 ice swimmers who regularly participated in cold water swims (training and competition), 40% of whom stated that they experienced fewer, more mild and shorter duration infections of the upper respiratory tract than before they started regular ice swimming. Brenke also observed that eight patients in a remote rural medical practice had a significant reduction in consultations for respiratory illnesses. Furthermore, Collier et al. [7] investigated the incidence and severity of upper airway infections in cold water swimmers with those within their immediate social circle (non-swimmers, partners and pool swimmers) and found that cold water swimmers had reported significantly fewer episodes of respiratory tract infections than their partners. Interestingly, the authors found no differences between cold water and pool swimmers. However, it is important to note that all three studies mentioned above relied on self-reported episodes of illness. 

Despite repeated claims about the benefits of swimming in cold water, there is mounting evidence that it can be potentially harmful. In the study by Collier et al. [7], the authors asked the participants to report episodes of upper respiratory tract infections each week over the course of the investigation. The authors noted a positive correlation between the prevalence and severity of upper respiratory tract infections and events of cold water exposure. While short-term exposure in cold water can certainly improve the activity of the immune system, repeated exposure without sufficient recovery may actually lead to a reduced immune function. Accordingly, Dhabhar [60] previously defined that short-term episodes of stress can last from minutes to hours, whereas chronic stress as hours, daily, weeks or months. Frequent swimming or prolonged immersion in cold water with persistent shivering during and after swimming may be classified in the latter category of physiological stress. In a recent study, Loria and colleagues [72] found that regular winter swimmers showed abnormal daily fluctuations in the concentration of cortisol. Furthermore, Dhabhar [60] described that this prolonged stress from chronic exposure could contribute to the dysregulation of the normal daily cortisol cycle contributing to the potential suppression of immune responses. Moreover, a possible contributing factor to the increased likelihood of upper respiratory tract infections may be through breathing in cold air and cooling the body surface, which may lead to bronchoconstriction and increased vasoconstriction in the nasal passages [13]. This is particularly relevant given that swimming training largely involves intermittent breathing rhythms, contributing to transient states of hypoxia [73]. Furthermore, it has been shown that exercise training above 80% of VO_2max_ contributes to lymphocyte apoptosis and a decrease in circulating white blood cells [74]. Coupled with disturbed daily cortisol rhythm, a greater oxygen consumption, and increased training intensity, it is possible that excessive exposure to cold leads to persistent physiological stress and this could lead to immunosuppression. Although several factors contribute to potential health benefits or risks, it is still unclear what the optimal dose may be, which is further compounded by individual variation in physiological response. 

## 12. Health Risks of Cold Water and Ice Swimming

Even if cold water swimming can provide a benefit in certain cases, the risks, especially with ice swimming, should not be disregarded. When swimming, it is important to be aware of potential cardiac and pulmonary risks that arise due to the cold exposure [75]. It is important to note that the thermoneutral temperature of humans is approximately 37 °C, and that prolonged immersion in water colder than 35 °C may produce hypothermia as body heat is lost to the environment [75]. The origin of this belief was formulated following the sinking of Titanic disaster [76] and supported by several observations made during the maritime conflicts of World War II. In recent years, research has been directed towards elucidating the pathophysiology of cold water immersion. It is has been theorized that there are four stages involved with cold water immersion that may result in incapacitation and rapid body warmth loss leading to hypothermia [77]. Each of the three stages of immersion are associated with special risks [77] (Table 2). The duration of these phases and the extent of the reactions caused in them vary considerably, depending on several factors, not least the water temperature.

## 13. Pathophysiology of Cooling and Swimming in Cold Water

Cold water and ice swimming require preparation, acclimatization and more importantly, cold water immersion experience and should be undertaken only with appropriate supervision in order to avoid injury or death. Even in the most experienced ice swimmers, cold water immersion carries risk of death. As outlined above the first stage is cold shock that is experienced on initial immersion [78] (Table 2).

The lungs contract in the first few seconds followed by uncontrollable hyperventilating and loss of breathing control [79]. The heart rate, and blood pressure and cardiac output increase rapidly increase with simultaneous peripheral vasoconstriction [4,80]. The dynamic response is initiated by peripheral cold receptors, peaking approximately 30 seconds after exposure and adapting over about two minutes [81]. The initial shock and loss of breathing control is where the swimmer is at greatest risk of drowning and death is it requires very little aspirated water to initiate the drowning process [82]. Experienced winter swimmers become more resistant to the cold shock reaction through conditioning and progressive adaptation of the body to the cold regularly and with increasing frequency and gradual lowering of temperatures [83]. It is crucial to adapt and acclimatize to the initial shock response as it is particularly hazardous, accounting for the majority of cold water immersion deaths [75]. 

Our understanding of the minimum rates of change in temperature within cold receptors, which are required to trigger cold shock, remains unclear. However, depending on several factors, a reaction can occur in water as warm as 25 °C [15]. Under artificially controlled conditions, respiratory drive as indicated by breathing rate has been shown to peak when immersed in a water temperature of between 10 and 15 °C. Interestingly, similar respiratory rates are observed, when immersed in water at 5 °C [19]. The lack of significant change in respiratory rate between 5 and 15 °C could be in part due to an autonomic change in breathing to conserve respiratory heat by decreasing expired air temperature and dead space ventilation [84]. 

Once a person is able to bring their breathing under control following the first two minutes of cold exposure, they will enter the second phase; short term exposure (Table 2). The physical challenges experienced during short-term immersion are predominantly related to the musculoskeletal impairment caused by neuromuscular cooling, interrupted of nerve conduction and increased nociceptive sensitivity (pain receptors) [85]. The arms, head and neck are particularly vulnerable due to their high surface to mass ratio and superficial location of major blood vessels [86]. As the cold temperature progressively cools deeper layers of muscle tissues, enzymatic activity is interrupted, the cellular metabolism slows, including the release and diffusion of calcium and acetylcholine [87]. In this context, muscle strength and fibre contractility depend on muscle temperature. Muscle force production has been show to decrease 4% to 6% per °C down to 30 °C [88]. Within the neuromuscular junction, as temperatures of the nerves drops below ~20 °C, electrical impulse and conduction are slowed down and the amplitude of the action potential is significantly reduced [89]. If the temperature of nerve drops lower to a temperature between 5 and 15 °C for 1–15 min, a nerve block can occur in both motor and sensory neurons [90]. This disruption in neuromuscular function can lead to a physiological state to peripheral paralysis and, as a result, may increase the risk of drowning. Furthermore, cold exposure has been shown to induce cognitive [91] decline and increase perceived stress [92], which may lead to a as a person not being able to adequately protect their airways [93,94]. 

Prolonged exposure to the cold may lead to hypothermia, but a hypothermic state usually does not arise for at least 30 min in healthy adults [95]. There is a greater variability between the low body core temperature and the onset of signs and symptoms of hypothermia. Hypothermia is a physiological state that affects cellular metabolism and function, may result in hemostasis, lactic acidosis, vascular insufficiency, cognitive impairment and arrythmia [96]. The progressing signs and symptoms depending on the core body temperature are summarized in Table 3 [82].

A further aspect is metabolic cost of shivering [97,98]. Shivering can generate heat at a rate of 10 to 15 kJ/min [98]. Acclimatization to cold develops over the course of about 10 days, and the primary change is a hypothermic type of response [98]. In winter swimmers, shivering was induced later during cooling (after 40 min) than in controls suggesting an important participation of non-shivering thermogenesis in the early thermogenic response [34]. It has been shown during cold water immersion that dynamic core temperature significantly contributed to the magnitude of metabolic heat production and that individual differences existed in central thermosensitivity [99].

## 14. The Risk of Hypothermia in Cold Water Swimming

As outlined above, the most significant risk of cold water swimming is hypothermia [41,43,100]. In a recent study, Knechtle et al. [43] observed elite ice swimmers in preparation for and an ice swimming event held at Lake Zurich (2.2 km in 4 °C cold water). During the event, the body core temperature of one of the swimmers dropped from 37 °C to 32 °C, 20 min after leaving the water [43]. 

However, it can be assumed that the drop in core body temperature in ice swimmers is less rapid than in pool swimmers with more subcutaneous fat [40,43]. Overweight people who are acclimatized to the cold water and have the appropriate experience are more likely to tolerate a longer stay in the cold water than people with little body fat tissue and those who are not acclimatized [40,79,102,103]. An experienced, overweight ice swimmer with a BMI > 35 kg/m^2^ and ~45% body fat never became hypothermic, even after several stays in ice-cold water [40]. Keatinge et al. [104] described a case of an Icelandic fisherman who survived in the ice-cold water, in part due more adipose tissue. After his boat sank and his two colleagues drowned within 10 min, he swam back to the shore in the 5 °C cold sea, taking him approximately 6 hours to complete [104]. 

Hypothermia has a lower risk of health damage from trained cold-water swimming. According to Tucker and Dugas [105], it also takes less than 30 min in cold water at 0 °C until the body temperature is so low decreases that hypothermia occurs [106]. According to various estimates, a person can survive in water at 0.3 °C for 45 min. In this case, death usually arises from cardiac stress rather than hypothermia itself [107]. However, exhaustion or loss of consciousness is expected to occur within 15 min. The consumption of alcohol before swimming in the winter should be avoided as this will accelerate the onset and progression of hypothermia [108].

An important aspect is the aspect of swimming intensity (e.g., in %VO_2max_) to maintain body core temperature while swimming in cold water (e.g., 10 °C) [109,110]. A cold-water swimmer covered a total swim distance of 15 km in water of 9.9 °C while swimming at a mean speed of 2.48 km/h [99]. The swimming speed of 2.48 km/h was equal to 0.69 m/s which corresponds to ~70%VO_2max_ [111].

Exercise might also have an effect of the decline in body core temperature during swimming in cold water [112]. It has been shown that physical exercise may predispose a person to a greater heat loss and to experience a larger decline in body core temperature when subsequently exposed to cold air [112]. However, a study investigating men while immersed in water of 3.6 °C showed that periods of work gave better chances of survival than continuous heat production by shivering in a well-insulated suit [113]. A further aspect is additional heat loss due to convective and/or conductive water flow [114]. Convective heat loss is the transfer of heat from a body to moving molecules such as air or liquid. Heat loss can occur by conduction of heat from the skin to the cold water around the body. It has been shown that mean skin heat loss was higher in wind and waves [115].

Caution is advised when swimming in pools and seas near the polar regions in winter. The chlorine added to the water in swimming pools and the salt in the sea water enable the water to remain liquid at sub-zero temperatures. Swimming in such waters is much more challenging and dangerous. The experienced winter swimmer Lewis Gordon Pugh swam near the North Pole at −1.7 °C and suffered a frostbite injury in his fingers. It took four months for him to regain the feeling in his hands [116].

## 15. Cardiac Risks

Recent work has suggested that a large proportion of cold water immersion deaths than previously thought can be attributed to arrhythmias resulting from activation of sympathetic and parasympathetic nervous system [117]. In certain individuals with pre-existing risk factors, the reactive autonomic response may provoke an arrythmia and result in cardiac arrest [75,117]. These thermoreceptors located in the skin, react to cold stimulus resulting in (sympathetic activation, cold shock) and in the oronasal area when immersed or by wave splashes (vagal stimulation, diving reaction) [118]. This has been termed ‘autonomous conflict’ [117], and is thought to play a crucial role in the etiology of dysrhythmias and arrhythmias in otherwise young and healthy individuals, especially in cases of prolonged cessation of breathing associated with immersion, diving or breath-holding [119]. However, for fatal arrythmias to occur, predisposing factors such as Long-QT-syndrome, coronary artery disease or myocardial hypertrophy are necessary for its development [117]. Although it is possible for non-fatal arrhythmias to cause death indirectly if physical incapacitation occurs, the individual aspirates water and drowns as a result [120,121]. A case report described a 12-year-old child with Long-QT-syndrome [122] and following a dive into cold water, an irregular cardiac rhythm occurred, leading to a further extension of the QT-interval, which resulted in a pulseless ventricular tachycardia of over 300 beats per min [122]. Many of these factors, including drug-induced Long-QT-syndrome, are acquired [117]. However, there is also a strong association exists between heritable long-QT syndrome and swimming [123]. Ishikawa et al. reported that 51 of 64 children with known Long-QT-syndrome developed significant arrhythmias while participating in swimming or diving [78]. 

It has been theorized that that swimming in ice-cold water may cause damage to the heart muscle [54]. In a field study with eight swimmers (three women, five men, 31−71 years) who were able to swim 500 m (four men, two women), 750 m (one woman) and 1000 m (one man) as part of an official ice swimming the hsTnI (high sensitivity troponin I) increased significantly [54]. HsTnI is widely used as a diagnostic tool in patients where a myocardial lesion, heart muscle damage, is suspected [57]. The NT-proBNP (N-Terminal pro-B-type natriuretic peptide) did not increase significantly, and no connection between hsTnI and NT-proBNP could be demonstrated. However, the increase in cardiac troponin could be associated with arrhythmias, an increased cardiovascular risk or even heart failure. In one case of an experienced ice swimmer who swam three ice miles in a row on their own, an increase in potassium in the blood could be demonstrated, which in turn could increase the risk of arrhythmias [41].

On the other hand, ice swimming increases the risk of sudden death, either due to the initial neurogenic cold shock response or due to a progressive decrease in swimming efficiency or hypothermia. In addition, people with manifest or unknown heart diseases are more likely to experience rhythm disturbances when immersed in ice-cold water [124]. Therefore, a step-by-step strategy to initiate and develop this leisure activity is recommended to promote and maintain acclimatization, protect against potential risks of cold-water exposure, and potentially take advantage of the promising health benefits [47].

## 16. Drowning

A reduction in performance and initial cardiorespiratory responses (cold shock response) to immersion in cold water are probably the main factors that contribute to drowning emergencies in swimmers [125]. In controlled laboratory settings, the first reactions to immersion or ‘cold shock’ were classified as the most dangerous period [81] and accounted for the majority of deaths from immersion in cold water [126]. These deaths were most often attributed to drowning, with the physiological reactions of wheezing and uncontrollable hyperventilation being triggered by the dynamic response of the cutaneous cold receptors and the small volume of water required to trigger the drowning process [82]. Generally, drowning leads to cardiac arrest within 2 min [49]. Quan et al. reported 1094 cases of drowning in open water [127], where in most cases (78%) the course was poor (74% deaths, 4% severe neurological consequences), and in the cases with good courses, 88% were submerged or submerged for less than 6 min. This percentage drops rapidly, with the risk of death or severe neurological impairment after discharge from the hospital being given as almost 100% if the duration of immersion exceeds 25–27 min [128]. However, if the water is cold, this time can be extended, whereby the current ‘record’ is 66 minutes immersion with almost complete recovery [129]. In such cases, the water temperature appears to be protective, and cases with underwater survival with minimal long-term consequences have only been described in water below 6 °C [130].

After a person is immersed in cold water, a cold shock reaction occurs that causes an uncontrollable heavy breathing (gasp). This is followed by a cold-induced hyperventilation [131] with a longer period of time with high-frequency breathing [63,69]. With prolonged exposure to cold, the respiratory rate is very high and it is assumed that the increased ventilation leads to progressive inefficiency when swimming [132] and respiratory muscle fatigue [125]. Inexperienced cold water swimmers could benefit from a graded and progressive cold water acclimatization program, in combination with mental training to improve control over the respiratory portion of the cold shock response [133]. Although it is possible to improve the initial cold shock response, drowning may still occur. Uncontrollable hyperventilation triggered by the acute exposure to cold leads to a reduction in cerebral blood flow with the result of disorientation and loss of consciousness [80]. 

It has been previously shown that an acute cold reaction may lead to metabolic acidosis, which is compensated for by an acute respiratory reaction [41]. In an ice float, metabolic acidosis with increased lactate and increased partial pressure of CO_2_ as well as a decrease in base excess and in HCO_3_ [63] occurred after each ice mile. Exhaling can cause water enter the airways, which can lead to drowning.

## 17. Death in Ice Swimming

So far, two deaths have been reported in an official ice swimming competition. In December 2018, a native and very experienced ice swimmer died in Russia after his race over 50 m dolphin. On the way to the warm-up area, he collapsed unexpectedly and died on the spot. The water was 0 °C cold, the air was −22 °C [134]. At the end of December 2019, a 51-year-old swimmer died after an official competition in Shaungyashan in China. The swimmer passed the medical examination before the start without problems, but felt uncomfortable during the 500 m long ice swim, was taken out of the water and transferred to the hospital where he died shortly afterwards [135].

## 18. Pulmonary Risks

Swimming in ice-cold water can lead to pulmonary problems [136,137,138,139]. The development of pulmonary edema is the most common problem [138,139]. A systematic review showed that there is a connection between water temperature and the occurrence of a SIPE (swimming induced pulmonary edema). The presence of the clinical symptoms cough, shortness of breath, foam and hemoptysis strongly suggests a SIPE during or immediately after swimming [136].

## 19. Conclusions

In summary, ice swimming is held as an official competition swim in various swimming routes and disciplines at water temperatures below 5 °C. Regular swimming training in cold water seems to have a positive effect on various systems such as the cardiovascular system, endocrine system, immune system and the psyche. However, cold water swimming still poses a significant health risk for inexperienced and untrained swimmers. It is recommended that in order to fully benefit from the metabolic and thermogenic effects of cold water swimming, a grade and progressive acclimatization program is required and preferably done under supervisor. That being said, cold water swimming (ice, cold and winter swimming) is an exciting new discipline and further research is required to fully understand the health benefits of its undertaking.

## Figures and Tables

**Table 1:** Benefits of cold-water swimming.

System	Effects	References
Cardiovascular system	Lowering blood pressure	[23,47,53,54]
Endocrine system	Decrease in triglycerides	[24,31,55]
	Increase in insulin sensitivity	[3,11,31,56]
	Decrease in norepinephrine	[53]
	Increase in cortisol	[57,58]
Psyche	Antidepressant effect	[8,59]
Immune system	Increase in leucocytes	
	Increase in monocytes	[27,28,60,61]
	Fewer infections	[7,13,62]

**Table 2:** Three stages of immersion in cold water.

Initial (Cold Shock)	First Three Minutes	Cooling of the Skin, Hyperventilation, Tachycardia, Gasp Reflex
Short-term	After three minutes	Superficial neuromuscular cooling
Long-term Circum-rescue collapse (afterdrop)	After 30 min Immediately before, during or after rescue	Hypothermia, later collapse Cardiac arrythmia, hemostasis, unconsciousness

**Table 3:** Symptoms of cold-water immersion according to core body temperature [101].

36 °C	Spontaneous shivering, rapid heart rate, rapid breathing rate
35 °C	Confusion, disorientation, decreased muscle coordination
34 °C	Amnesia
33 °C	Cardiac arrhythmias, poor perfusion of the skin
33–30 °C	Clouding of consciousness, spontaneous shivering stops, rigid muscle tone
30 °C	Stupor or unconsciousness, diminished respirations, poor muscle tone, hypotension
28 °C	Ventricular fibrillation, near absent respiration, vital signs near absent
25 °C	Cardiac arrest, death
